# Endometriosis Susceptibility to Dapsone-Hydroxylamine-Induced Alterations Can Be Prevented by Licorice Intake: In Vivo and In Vitro Study

**DOI:** 10.3390/ijms22168476

**Published:** 2021-08-06

**Authors:** Chiara Sabbadin, Alessandra Andrisani, Gabriella Donà, Elena Tibaldi, Anna Maria Brunati, Stefano Dall’Acqua, Eugenio Ragazzi, Guido Ambrosini, Decio Armanini, Luciana Bordin

**Affiliations:** 1 Department of Medicine—Endocrinology, University of Padova, 35121 Padova, Italy; ChiaraSabbadin@libero.it; 2 Department of Women’s and Children’s Health, University of Padova, 35121 Padova, Italy; alessandra.andrisani@unipd.it (A.A.); guido.ambrosini@unipd.it (G.A.); 3 Department of Molecular Medicine-Biological Chemistry, University of Padova, 35131 Padova, Italy; gabriella.dona@hotmail.com (G.D.); elena.tibaldi@unipd.it (E.T.); annamaria.brunati@unipd.it (A.M.B.); 4 Department of Pharmaceutical and Pharmacological Sciences, University of Padova, 35131 Padova, Italy; stefano.dallacqua@unipd.it (S.D.); eugenio.ragazzi@unipd.it (E.R.)

**Keywords:** endometriosis, dapsone, DDS-NHOH, red blood cell, glycyrrhetinic acid

## Abstract

Endometriosis, an estrogen-dependent chronic gynecological disease, is characterized by a systemic inflammation that affects circulating red blood cells (RBC), by reducing anti-oxidant defenses. The aim of this study was to investigate the potential beneficial effects of licorice intake to protect RBCs from dapsone hydroxylamine (DDS-NHOH), a harmful metabolite of dapsone, commonly used in the treatment of many diseases. A control group (CG, *n* = 12) and a patient group (PG, *n* = 18) were treated with licorice extract (25 mg/day), for a week. Blood samples before (T_0_) and after (T_1_) treatment were analyzed for: i) band 3 tyrosine phosphorylation and high molecular weight aggregates; and ii) glutathionylation and carbonic anhydrase activity, in the presence or absence of adjunctive oxidative stress induced by DDS-NHOH. Results were correlated with plasma glycyrrhetinic acid (GA) concentrations, measured by HPLC–MS. Results showed that licorice intake decreased the level of DDS-NHOH-related oxidative alterations in RBCs, and the reduction was directly correlated with plasma GA concentration. In conclusion, in PG, the inability to counteract oxidative stress is a serious concern in the evaluation of therapeutic approaches. GA, by protecting RBC from oxidative assault, as in dapsone therapy, might be considered as a new potential tool for preventing further switching into severe endometriosis.

## 1. Introduction

One of the most common gynecological pathologies in reproductive age women is endometriosis, characterized by the presence of endometrial-like tissue in the uterine cavity [1,2].

Both heme and cellular debris contribute consistently to the formation of local [3] and systemic [4] inflammation status, thus promoting the increased production of reactive oxygen species (ROS) and reactive nitrogen species, cytokines, growth factors, and prostaglandins [5]. 

The presence of oxidative stress status markers in circulating red blood cells (RBCs) has been recently pointed out as a systemic feature of endometriosis [4]. Due to their fundamental role in oxygen transport, RBCs are particularly exposed to the oxidant threat, which represents a limiting factor of the lifespan of RBCs because no new proteins can be synthesized [6]. Therefore, oxidative-related damage is critical in the regulation of RBC’s proper functioning and aging. 

Besides ensuring deformability, which is determined by membrane protein-protein and lipid-protein interactions, membranes also provide cellular ion exchange and the expression of aging-related epitopes for RBC senescence recognition and removal.

In the RBC membrane, one of the most important integral proteins is protein band 3 (or anion exchanger, AE1), a 100 kDa protein with 12–14 transmembrane segments, mainly involved in the maintaining of the biconcave-shape and the CO_2_/HCO_3_^−^ homeostasis through chloride and bicarbonate (Cl^−^/HCO_3_^−^) anion exchange [7]. The presence of phosphorylatable residues in the cytoplasmic domain (including both the *N*- and *C*-terminal ends of the molecule) provides band 3 the peculiarity of being considered as a redox stress sensor [8] in many prooxidant disorders [8,9,10], such as in glucose-6-phosphate dehydrogenase deficiency (G6PDd) [10,11]. The band 3 Tyr-P level regulates many of the physiological processes in RBCs, from glycolysis [12] to morphology [13], but it is also involved in erythrocyte aging [11,14] and antibody recognition [15].

Dapsone (DDS) is an aniline compound commonly used for many indications [16,17], including the treatment of leprosy, varied skin conditions, *Pneumocystis carinii* infection, and a variety of immuno-related conditions [18,19]. Unfortunately, DDS shares a well-documented toxicity, related to its routes of biotransformation [20,21] leading to the formation of dapsone hydroxylamine (DDS-NHOH), the powerful oxidizing dapsone metabolite [20,22]. In in vitro studies, DDS-NHOH has been demonstrated to shorten RBC lifespan through the progressive oxidative alteration pathway starting from methemoglobin formation, glutathione oxidation, [22,23,24,25], and band 3 high molecular weight aggregates (HMWA) [26], which leads to autologous antibody recognition [24].

Among the antioxidants assumed with the diet, such as vitamins, carotenoids, and minerals, which have been shown to contribute to maintaining the redox homeostasis, glycyrrhizin, the glycoside extracted from roots of the liquorice, known for its characteristic sweetness (about 30–50 times sweeter than sucrose), is widely used in the treatment of many diseases, such as chronic hepatitis [27], erythrodermic psoriasis [28], a variety of human viruses such as avian infectious bronchitis virus [29], HIV [30,31], and SARS-CoV-2 [32,33], as a few examples. Orally administered glycyrrhizin is metabolized by intestinal bacteria into 18β-glycyrrhetinic acid (GA) [34], a pentacyclic triterpenoid, whose structure is similar to those of the mineralocorticoid and glucocorticoid hormones secreted by the adrenal cortex. In in vitro experiments in human RBCs, GA prevented oxidative-induced alterations, greatly reducing both band 3 Tyr-P and band 3 high molecular weight aggregate (HMWA) formation [35]. 

The aim of this study was to evaluate the effect of the licorice intake on the oxidative stress generated by DDS-NHOH in RBCs from PG by monitoring band 3 Tyr-P levels and HMWA as parameters for the detection of RBC membrane denaturation. In addition, we analyzed the state and the activity of cytosolic carbonic anhydrase (CA), to investigate if GA could mitigate DDS-NHOH side-effects involving this enzyme, a useful parameter of the potential worsening of RBC oxidative status [36].

## 2. Results

### 2.1. Evaluation of the RBC Oxidative Status (Diamide) and Response to DDS-NHOH before Licorice Intake

**Figure 1:**
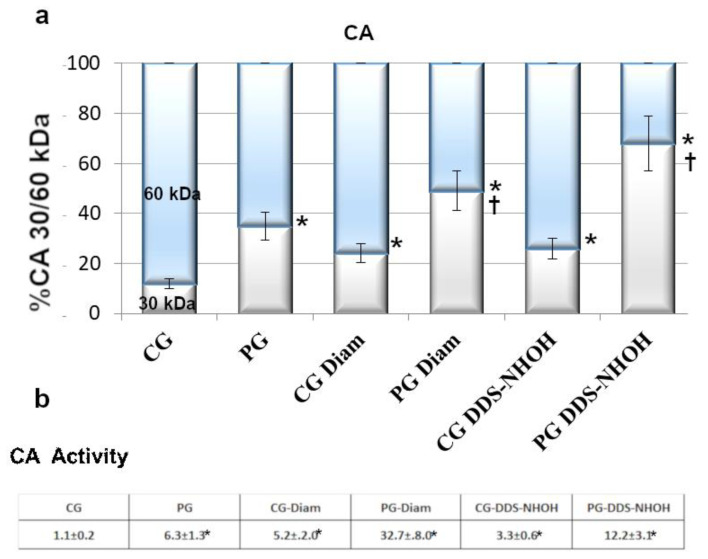
Fresh blood was collected from CG and PG RBCs (isolated as described in the Methods section) and was incubated with and without 1.5 mM diamide or 0.3 mM DDS-NHOH. (**a**) Diluted cytosol from 1 μL of packed cells, underwent Western blotting in non-reducing conditions. Bands immunostained with anti-CA antibodies were densitometrically analyzed, and the sum of the 30 and 60 kDa bands was arbitrarily calculated as 100%, taking into account that amount of proteolytic 30 kDa bands accounts for half the larger bands. Values were expressed as the means ± SD of *n* = 12 CG and *n* = 18 PG patients. * *p* < 0.001, comparison of CA 30 kDa isoform before and after treatments within groups, Student’s *t*-test for paired data; † *p* < 0.001, comparison of the 30 kDa band between CG and PG groups, in both experimental conditions (diamide and DDS-NHOH treatments), Student’s *t*-test. (**b**) CA activity: 300 μL of diluted cytosol from CG or PG RBCs, previously incubated with and without 1.5 mM diamide or 0.3 mM DDS-NHOH, were assayed for activity as described in the Methods section. The activity was calculated as the ratio to activity observed in untreated CG (chosen as arbitrary comparison unit, experimentally determined as 1 ± 0.23, mean value ± SD). Data show the means ± SD of *n* = 12 CG and *n* = 18 PG patients. * *p* < 0.001, comparison of CA activity to CG, before and after diamide treatment, Student’s *t*-test for paired data.

Cytosolic compartments of PG RBC showed much higher monomeric CAII isoform (30 kDa) than CG RBC (35 ± 3.7% compared to 12 ± 3%, respectively, *p* < 0.001). Being the activity of CA strictly depending on its monomerization [36], a CA activity assay was performed, with PG RBC exhibiting almost six times higher values compared to CG RBC (Figure 1). When RBCs were treated with diamide, the amount of the 30 kDa band of CA increased to 24% and 49%, in CG and PG RBC, respectively, (*p* < 0.001), with a parallel activity increase (5.2 ± 2 in CG and 32.7 ± 8.0 in PG, *p* < 0.001). In addition, in the presence of DDS-NHOH, if the process of formation of the 30 kDa isoform was more evident (26% in CG, 68% in PG), the correspondent values of CA activity were not so drastically increased as expected (3.3 ± 0.6 and 12.2 ± 3.1 in CG and PG, respectively).

### 2.2. Effect of Licorice Intake on the Membrane and Cytosol Oxidative Status

**Figure 2:**
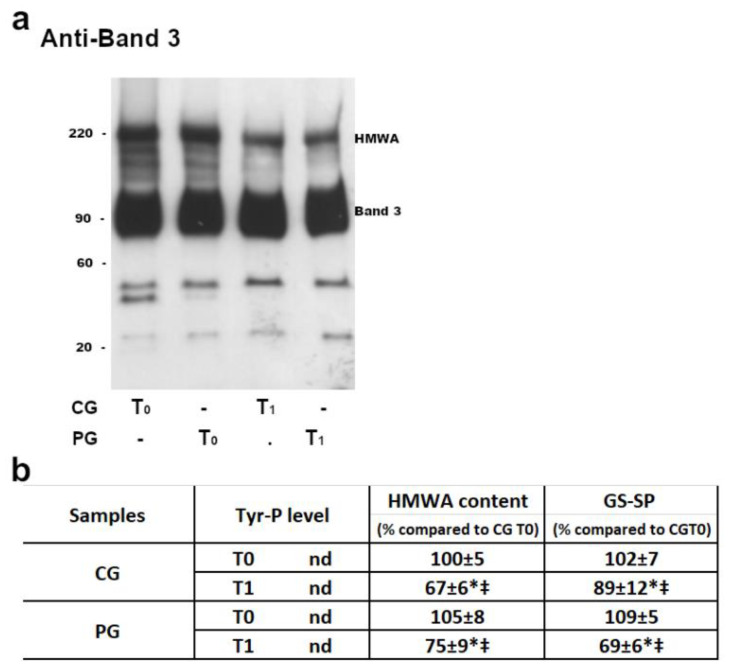
The membranes (10 μg) obtained, as described in the Methods section, were analyzed by Western blotting in non-reducing conditions (panel **a**) and immunostained with anti-band 3 P-Tyr antibodies or in non-reducing conditions and immunostained with anti-band 3 (panel **a** and **b**), anti-P-Tyr or anti-GSH antibodies (panel **b**). For each immunostaining, bands corresponding to the relative proteins were densitometrically estimated and statistically analyzed (panel **b**). The Tyr-P value of both CG and PG RBCs were undetectable. The band 3 HMWA or GSH values were calculated as the ratio to band 3 HMWA or GSH obtained in basal (T_0_) samples of CG (chosen as arbitrary comparison unit, experimentally determined as 100 ± 5% and 102 ± 7%, respectively). Data shows the means ± SD of *n* = 12 CG and *n* = 18 PG patients. Comparison from respective baseline values: * *p* < *0*.001, Student’s *t*-test for paired data. Comparison CG vs PG: ^‡^ *p* < *0*.001, Student *t*-test for unpaired data.

In both CG and PG, RBC membranes at T0 (before licorice intake) showed a similar pattern of B3 Tyr-P levels (which remained practically undetectable) and HMWA content (Figure 2, panel b).

**Figure 3:**
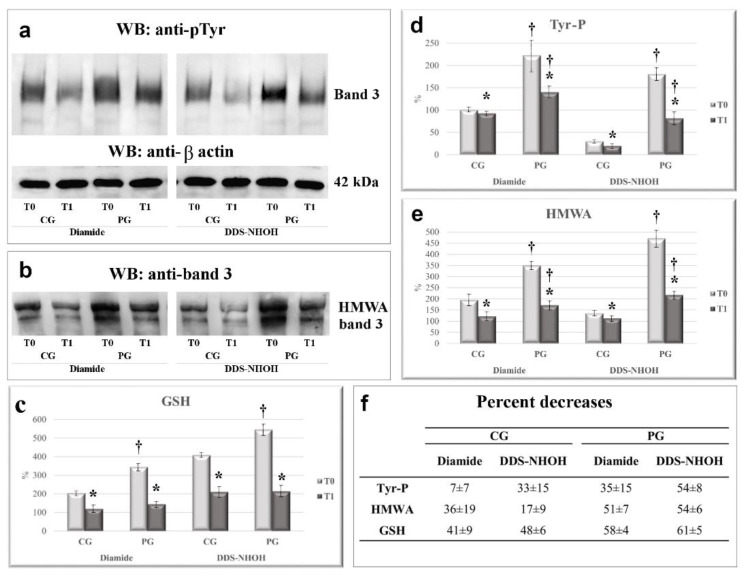
The effect of 1-week licorice intake on the Tyr-P level (**a**), HMWA, (**b**) and membrane GSH contents (**c**), following diamide or DDS-NHOH stimulation, in the CG and PG groups. RBCs from CG and PG were incubated with 1.5 mM diamide or 0.30 mM DDS-NHOH. The membranes (10 µg) obtained, as described in Methods, were analyzed by Western blotting and immunostained with the anti-P-Tyr antibody and then with anti-β actin as a loading control (Panel **a**). The membranes were also analyzed in non-reducing conditions and immunostained with anti-band 3 (Panel **b**) or anti-GSH (not shown) antibodies, in order to evidence the band 3 high molecular weight aggregate (HMWA) and bound glutathione, respectively. The figure is representative of the study population. For each immunostaining, bands corresponding to the relative proteins were densitometrically estimated and statistically analyzed GSH values of diamide or DDS-NHOH treated RBCs were calculated as the ratio percentage to GSH levels obtained in the basal samples of CG at T_0_ (chosen as an arbitrary comparison unit, experimentally determined as 101 ± 3%) (Panel **c**). The Tyr-P value of diamide—or DDS-NHOH—treated RBCs before (T_0_) and after a week of licorice intake (T_1_) was calculated as: Tyr-P% = (Tyr-P(x)/Tyr-P(CG) diamide T_0_%, with the Tyr-P value obtained in diamide samples of CG at T_0_ chosen as an arbitrary comparison unit (experimentally determined as 100 ± 5%) (Panel **d**). The band 3 HMWA of diamide or DDS-NHOH treated RBCs were calculated as the ratio percentage to band 3 HMWA obtained in the basal samples of CG at T_0_ (chosen as an arbitrary comparison unit, experimentally determined as 100 ± 4 and) (Panel **e**). (Panel **f**) average percent decrease for each parameter, referring to the corresponding values at T_0_, were calculated and reported. Data show the means ± SD of *n* = 12 healthy subjects (CG) and *n* = 18 patients (PG). Comparison from the respective T_0_ values: * *p* < 0.005, Student’s *t*-test for paired data. Comparison CG vs PG: † *p* < 0.001, Student *t*-test for unpaired data.

In the presence of diamide, RBCs from PG showed a much higher Tyr-P level in RBC membranes compared to that from CG (221 ± 26 and 100 ± 9 for PG and CG membranes, respectively, *p* < 0.005). Similarly, membrane band 3 HMWA content also increased to double the basal amount in CG but reached almost four times the basal value in PG (196 ± 19 and 349 ± 45, for membranes from CG and PG, respectively, *p* < 0.001) (Figure 2, panel a, lanes T_0_ and Figure 3, panels a and b, compare CG and PG at T_0,_ diamide) [4]. When diamide was replaced by DDS-NHOH, the average increment in the Tyr-P level was almost 6 times in RBCs from PG compared to CG (*p* < 0.001) (Figure 3, panel a). Similarly, in PG, DDS-NHOH treatment induced a higher increase in band 3 HMWA content (average PG increase of about 471 ± 37 % compared to CG, *p* < 0.001), much higher than that evidence in the RBC membranes of CG (196 ± 35 and 135 ± 29 for diamide and DDS-NHOH treatment, respectively, *p* < 0.01) (Figure 3, panels a and b, compare CG and PG at T_0,_ DDS-NHOH). 

The increased oxidative status of the membrane was also evaluated by glutathionylated protein content (average increase of about 145% and 140% for diamide and DDS-NHOH, respectively, *p* < 0.001) (panel c).

After 1 week of licorice intake (T_1_), RBCs from both groups were reanalyzed and the HMWA anti-Tyr-P and anti-GSH content from PG were compared to those from CG (Figure 2). 

Results showed that both the HMWA and protein-bound GSH (GS-SG) contents were reduced after licorice intake (T_1_), both in CG (with an average decrease of about 30% in HMWA and 10% in GS-SP) and PG (average decrease of 25% in HMWA, but almost 40 % in the GS-SP). Either in CG or PG, no alterations were detectable in the Tyr-P level.

Interestingly, at T_1_, the PG RBCs also showed a net reduction in both diamide- and DDS-NHOH-induced alterations compared with their own values at T_0_, with an average decrease ranging from 35 ± 15% (diamide-induced Tyr-P level) to 61 ± 5% (decrease of DDS-NHOH-induced glutathionylation) (panel f). All the other parameters ranged between these two values, with a reduction higher than 50%. CG RBCs showed a slight decrease only in diamide-induced Tyr-P values (7 ± 7%), but in all the other parameters the average decrease ranged from a minimum of 17 ± 9% (DDS-NHOH-induced HMWA formation) to a maximum of 48 ± 6% (the decrease in membrane glutathionylation was induced by DDS-NHOH). The variations of all parameters observed between T_0_ and T_1_ were statistically different, both in the CG and PG groups (*p* < 0.005). Also, the average values were statistically different between the CG and PG groups (*p* < 0.001) in all conditions (panels a–c), except for DDS-NHOH-induced glutathionylation, because, following licorice intake (T_1_), the higher level of glutathionylated protein in PG RBCs was almost completely lowered, thus resembling the level in the CG RBCs (panel c, T_1_).

### 2.3. Licorice Intake and CA Monomerization and Activity in RBC Cytosol

The cytosolic oxidative status was also evaluated, and the monomerization and activity of CA and the variation of GSH contents were compared between the two groups in the presence of diamide or DDS-NHOH at T_0_ and T_1_. As expected, in PG, at T_0_ the monomeric form of this enzyme, representative of increased oxidation [26,36], was much higher compared to that of CG (35 ± 5 % of the 30 kDa isoform in PG compared to 12 ± 4 % present in CG, *p* < 0.001), and, consequently, also the CA activity was by far higher (Figure 2, *p* < 0.001).

What is interesting is that, following licorice intake, net decreases of both monomerization and activity were observed. By evaluating the average decrease in both values between T_1_ and T_0_, at T_1_ the percentage of CA activity reduction was 8.5 ± 7 and 50.7 ± 4.3, in CG at basal or DDS-NHOH conditions, respectively (*p* < 0.005). In PG, CA activity reduction was 61.7 ± 16 and 79.1 ± 8.7, at basal or DDS-NHOH conditions, respectively (*p* < 0.005) (Table 1).

When the cytosol was analyzed for the GSH content, DDS-NHOH treatment induced a drop in the total glutathione content of about a mean value of 0.85 ± 0.08 in PG RBCs (Table 1, panel b, T_0_). In CG only, a slight decrease in the glutathione content was observed after both diamide and DDS-NHOH treatments, 0.08 ± 0.03 and 0.20 ± 0.01, respectively, thus confirming that the band 3 Tyr-P level and HMWA formation involved cell GSH-related anti-oxidant defenses, with the depletion of cytosolic pools to implement the membrane protein glutathionylation. Also, in this case, licorice intake induced a net reduction of the GSH lost, as indicated by the lowering of ΔGSH values in both CG and PG (Table 1).

### 2.4. Correlation between Plasma GA Content and Reduction of RBC Oxidative Parameters

**Figure 4:**
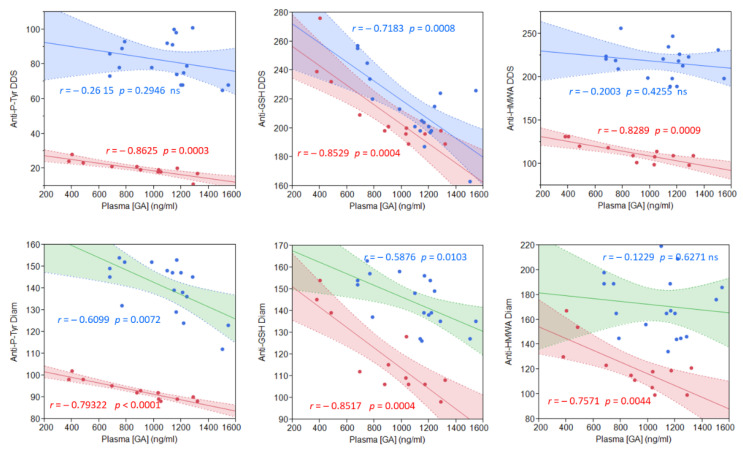
Correlation between the plasma GA concentration and measured parameters, after 1-week of licorice treatment, in both CG (red points) and PG (blue points) groups. Upper graphs: stimulation with DDS-NHOH. Lower graphs: basal values (not stimulated). For each regression line, Pearson’s correlation coefficient *r* is indicated, together with the corresponding *p*-value. To obtain the plasma GA concentration, an HPLC–MS full scan method was used as described in the Methods.

To assess if these licorice intake-related improvements and GA metabolites were correlated, we quantified the plasma GA content following the licorice intake (Figure 4). Plasma GA concentrations ranged from a minimum of 484 to1546 ng/mL in both groups, representative of great subjectivity in metabolizing licorice to yield GA. No significant difference was found between the mean GA plasma concentrations in the two groups (886.7 ± 329.3 ng/mL in CG vs. 1087.8 ± 261.6 ng/mL in PG; *p* = 0.0734). What is noteworthy is that we found a direct proportionality between the plasma GA content and a reduction in both diamide and DDS-NHOH-induced alterations (Figure 4), which resulted in highly significant for all parameters in CG, and less in PG (see correlation coefficients reported in Figure 4).

The highly significant proportionality between the plasmatic GA content and the reduction of diamide-induced alterations was consistent with previous in vitro observations [35], but only for the Tyr-P parameter. Compared to DDS-NHOH, GA was not so efficacious in reducing diamide-induced membrane glutathionylation and HMWA formation. On the contrary, the GA plasma concentration better fitted with the improvements of both membrane and cytosol parameters following DDS-NHOH treatment, thus evidencing those alterations induced by the two compounds were different and, so, differently affected by GA. 

## 3. Discussion

In this study, we investigated the effect of licorice intake on RBC improvements towards oxidative stress in endometriosis. 

Dapsone-induced hemolytic anemia is closely related to erythrocyte membrane alterations, leading to premature cell removal, which can occur both extra-vascularly (witness hyperbilirubinemia), or intravascularly by dapsone-induced cell fragility. All hematological side effects reported for dapsone therapy are due to the N-hydroxy metabolite of the drug, dapsone hydroxylamine (DDS-NHOH) [20,25,37].

These alterations should be taken into account in choosing therapy for endometriosis patients. Endometriosis is a chronic inflammatory disease with a genetic, epigenetic, and environmental background [38]. It has been recently shown that the presence of endometriosis susceptibility genes whose wide variations of penetrance would be seriously influenced by phenotypic alterations [39]. Environmental changes, such as iron overload during menstruation, can induce a Fenton-mediated oxidative assault, which would affect DNA hypermethylation and chromatin remodeling, thus stressing gene instability by introducing point mutations and/or DNA single- and double-strand breaks, all leading to a significant increase in cancer risk [39]. For these reasons, redox and inflammatory modifications, which can accumulate in endometriosis patients, may be not only at the origin of endometriosis but also responsible for the further development/worsening of the disease [39].

In this study, we addressed the potential effect of licorice in ameliorating PG RBC tolerance to dapsone treatments. We have previously demonstrated that GA, one of the licorice intestinal metabolism products, was able to prevent diamide-induced band 3 Tyr-P levels and HMWA formation, as well as band 3 proteolytic degradation in in vitro experiments performed with normal RBCs [35]. To investigate if this important GA shielding effect could be efficacious also in endometriosis to lower potential oxidant injuries, we analyzed the same parameters in RBCs after volunteers were given one week of licorice intake. Interestingly, all parameters resulted in positively affected PG RBCs, with net reductions in DDS-NHOH-induced alterations ranging from 35 to 61%, compared to T_0_. That this effect was due to the GA licorice component was confirmed by the correlation between the plasma GA content and diamide, used as a reference, with its effects amply studied and described in previous studies [35,40], or DDS-NHOH effects. Only with diamide, for bound GSH and HMWA formation parameters, it seemed that weak a correlation was present in PG, but not in CG. This could be explained by the fact that diamide-induced alterations are different from those by DDS-NHOH. Diamide is known to induce disulfide bond formation, thus clustering membrane band 3 and leading to HMWA formation. Band 3 is normally distributed between detergent soluble (66%) and detergent-insoluble (33%) fractions of RBC membranes, and following diamide treatment, band 3 aggregated in HMWA increased only in the detergent soluble fraction [41]. On the contrary, DDS-NHOH induces a complete rearrangement of HMWA, which starts at the soluble fraction but slowly migrates to increase the insoluble counterpart [26]. 

Among the cytosolic enzymes, an important role is played by CA, a metallo-enzyme, converting CO_2_ to HCO_3_- and H +, which regulates many physiological processes such as acid–base balance homeostasis, respiration, carbon dioxide, ion transport, and bone resorption [42]. To date, their biological functioning has not been clarified, but recent evidence has pointed out how abnormal levels or activities [34] of many CA isoforms were associated with different diseases such as cancer (overexpression of CA IX/XII due to the hypoxia cascade activation), epilepsy (abnormal levels/activities of brain CA isoforms), and obesity (dysregulation of the mitochondrial isoforms CA VA/B) [42]. 

In human RBCs, the upregulation or high activity level of CA 2, the main isoform [36], has been related to glaucoma [42], and for this its functioning could represent an important parameter to be evaluated, mainly due to the recent finding showing an oxidative-related net increase of CA 2 activity in endometriosis patients. In fact, by increasing oxidative conditions, CA 2, normally present as an inactive dimer, can be activated following a monomerization process [36]. In the present study, the mean CA activity from PG was about 30 times higher than that from CG with a mean monomerization three times superior compared with CG, and 5 times lower GSH (ΔGSH). These data identify CA as an important parameter in the evaluation of the oxidative status in endometriosis, as well as a novel paradigm in the prevention of potential clinical complications. 

In PG, one week of GA intake succeeded in considerably reducing both untreated and, much more interestingly, DDS-NHOH treated RBC effects on CA, with 5 times activity reduction and 3 times less monomerization, whereas in CG, both CA activity and monomerization, and GSH drop, returned to the level of the DDS-NHOH untreated RBC. 

GA, a licorice metabolite, mitigates DDS-NHOH-induced side effects by lowering membrane sensitivity to oxidative stress and preserving cell GSH content. 

For a long time, licorice has been considered in many natural medical resources, and these findings emphasize how GA can protect RBCs from strong oxidant-induced denaturation, thus preventing risks from extensive and prolonged exposure to oxidative stress in impaired anti-oxidant conditions.

## 4. Materials and Methods

### 4.1. Materials

Reagents were purchased from Sigma (Milan, Italy), and an anti-phospho-tyrosine (P-Tyr) (clone PY20) mouse monoclonal antibody was obtained from Biosource-Invitrogen (Camarillo, CA, USA). Anti-mouse secondary antibody conjugated with horseradish peroxidase (HRP) was obtained from BioRad Laboratories (Irvine, CA, USA), and DDS-NHOH from Toronto Research Chemicals Inc. (North York, ON, Canada).

### 4.2. Participants

Between December 2011 and December 2016, patients, presenting with pelvic pain and an ultrasonographically that identified an adnexal ovarian mass, were referred to our endometriosis care unit for laparoscopy. Only women classified as having endometriosis by histological examination of surgical specimens were put in the endometriosis group (*n* = 18, aged 34.1 ± 8.5 years). Following surgery, the stage of the disease was defined according to the classification system of the revised American Society for Reproductive Medicine (rASRM) as stage I (minimal, *n* = 4), stage II (mild, *n* = 7), stage III (moderate, *n* = 4), and stage IV (severe, *n* = 3).

Patients met the following criteria: no hormone therapy for at least 3 months; regular menstruation; non-smoker; no signs of other inflammatory disease (as assessed by leucocytes, body temperature, or other specific symptoms). Fresh blood was collected from patients undergoing laparoscopy and also from a group of 12 volunteers, mean age 34.9 (SD 9.2) years, whose clinical and ultrasound tests identified as being healthy (Table S1). 

Clinical data and peripheral blood samples were collected from both PG and CG subjects only after explaining the objectives of the study and obtaining signed informed consent, according to the Italian Law for Privacy 675/96 prior to enrolment.

All participants were asked to take licorice sweets containing licorice extract up to 25 mg /day, a dose by far lower than what is recommended by the Scientific Committee which considered it prudent that regular ingestion should not exceed 100 mg/day [43].

This study was conducted in accordance with the ethical standards of the Ethics Committee for Research and Clinical Trials of our University (Em. n. 7, 13 February 2012) and in accordance with the Helsinki Declaration.

#### 4.2.1. Treatment of Erythrocytes

RBCs were pelleted at 3750× *g* for 3 min. After removal of the supernatant, packed RBCs were washed three times at 3750× *g* for 3 min in 5 volumes of Dulbecco’s Phosphate Buffered Saline (D-PBS) to avoid contamination by leucocytes and platelets. Packed cells (50 µL) were suspended (at 20% hematocrit) in D-PBS and treated at 35 °C for 30 min in the absence (Basal) or presence of 1.5 mM diamide (dissolved in D-PBS) (Diamide), or 0.3 mM DDS-NHOH dissolved in acetone (DDS-NHOH).

After washing, RBCs underwent hemolysis in 1.5 mL of hypotonic buffer (5 mM sodium phosphate, pH 8, 0.02 % sodium azide (NaN_3_), 30 µM phenylmethylsulphonyl fluoride (PMSF), 1 mM sodium orthovanadate, and a protease inhibitor cocktail) [24].

Membranes were separated from the cytosol by centrifugation (16,100× *g* for 20 min in an Eppendorf centrifuge) and washed once in a hypotonic buffer. Aliquots of membranes and the cytosol were analyzed by Western blotting in reducing or non-reducing conditions and immunostained with appropriate antibodies. 

#### 4.2.2. Determination of GSH 

Total glutathione was determined according to [10]. Briefly, 10 µL of cytosol obtained from differently treated erythrocytes were added to 2 mL of a reaction mixture containing 1.9 mL of phosphate 0.1 M/ EDTA 0.6 mM buffer, pH 7.4, 30 µL of 5,5′-dithio-bis(2-nitrobenzoic acid) (DTNB) 10 mM, 100 µL of NADPH 5 mM, and 10 µg glutathione reductase (GR), and analyzed spectrophotometrically at 412 nm. The total decrease of glutathione content after diamide or DDS-NHOH treatment (ΔGSH) was expressed as 1-GSH (Diam or DDS-NHOH)/GSH(Basal) [24].

#### 4.2.3. Esterase Activity Assay

The activity of CA was assayed in RBC cytosols by following the change in absorbance at 348 nm from the 4-nitrophenylacetate (NPA) to the 4-nitrophenylate (PNP) ion over a period of 10 min at 25 °C with a spectrophotometer (CHEBIOS UV–VIS), according to [36]. The enzymatic reaction was carried out in a total volume of 3.0 mL, containing 1.4 mL 0.05 M Tris–SO_4_ buffer (pH 7.4), 1 mL 3 mM NPA, 0.5 mL H_2_O, and 0.1 mL diluted cytosol. A reference measurement was obtained by preparing the same cuvette with a sample solution in the absence of incubation. One unit of CA activity was defined as the amount of enzyme which catalyzes the formation of 1 pmol PNP/min in standard conditions of incubation. The following formula incorporating the extinction coefficient was used to calculate: CA units × 10^−3^/µL packed RBC = OD × sample dilution factor/(min × 667), with an extinction coefficient of 667 [28,35]. The decrease following the licorice intake (ΔCA activity %) was calculated as: (1- activity T_1_/ activity T_0_)%.

#### 4.2.4. HPLC–MS Plasma Analysis

To obtain a metabolic profiling of plasma, an HPLC–MS full scan method was used, according to [44]. A Varian MS 500 equipped with a Prostar 430 autosampler and binary chromatograph 212 series (Varian, Palo Alto, CA, USA), was used as the HPLC–MS system. An Agilent (Milan, Italy) Eclipse XDB C−8 column (2.1 × 150 mm 3.5 μm) was used as a stationary phase. The mobile phase was composed of solvent A (acetonitrile with 0.5% acetic acid) and solvent B (water with 2% formic acid). Linear gradients of A and B were used as follows: 0 min, 10% A; 20 min, 85% A; 21 min, 100% A, 21.30 min, 10% A; 27 min, 10% A. The flow rate was 200 μL/min and the injection volume was 10 μL. The mass range explored was 50–1000 *m/z*. The mass spectra were recorded both in positive standard mode and in turbo data depending scanning (tdds) mode that allows the elucidation of the fragmentation patterns of the detected ions. Collected plasma samples were centrifuged (13,000× *g* for 10 min) and directly injected in the HPLC. Each HPLC–MS data set was processed with the MZmine 2.9 software; from the raw data files, a data set composed of 102 variables was obtained. The Median Fold Change normalization was applied to take into account the effects of sample dilution. Data were log-transformed and mean-centered.

### 4.3. Statistical Analysis

Data are expressed as the mean ± SD. Differences between the groups were compared with the Student’s *t*-test (two-tailed). Comparisons before and after treatment with licorice within each group of subjects were obtained with the Student’s *t*-test for paired data. The paired *t*-test was also used to compare values before and after DDS-NHOH treatment. 

Relationships between pairs of variables were tested by least-squares linear regression. Pearson’s correlation coefficient *r* was used to quantify the strength of relationships. The statistical significance of *r* was determined using a *t*-test (two-tailed). 

A *p*-value < 0.05 was considered as statistically significant.

## 5. Conclusions

Endometriosis RBC membranes are characterized by high oxidative levels, which impairs the RBC response to a potential high oxidant therapy, such as in the case of DDS, commonly used for the treatments of leprosy, malaria, and autoimmune diseases. DDS-NHOH, a DDS metabolite, exasperates the oxidative status of the patients’ RBCs. The resulting condition leads to a premature RBC removal from circulation as an index of reduced RBC life-span due to the overwhelming oxidative assault [24]. It is also involved in a potential further worsening of patients’ conditions by increasing the oxidative stress which, in turn, may also trigger genetic/epigenetic cell transformation [38,39]. The DDS-NHOH-related further drop in the total glutathione content was also responsible for the serious increase of CA activity, thus bringing new concerns for the development of further complications, such as glaucoma [45].

After licorice intake, the GSH loss was clearly reduced, showing a net improvement of the cell anti-oxidant defenses, as confirmed by the related reduction of CA monomerization and activation. A similar protective effect of GA also mitigated DDS-NHOH induced side effects by lowering membrane sensitivity to oxidative stress and preserving the cell GSH content. 

In conclusion, the results demonstrate that licorice intake prevented/ameliorated the oxidative stress generated by a strong oxidizing agent, a byproduct of a commonly used therapy, in RBCs already seriously struggling with an endometriosis-related inflammatory status—but it is far from being an endometriosis therapy. Our study represents a promising pilot study that would request further investigations to better evaluate licorice potential in inflammatory diseases.

## Figures and Tables

**Table 1:** The effect of 1-week licorice intake on CA activity and monomerization and ΔGSH in the cytosol of RBCs, in the presence of diamide or DDS-NHOH in in vitro treatment. Panel **a**: CA activity and monomerization values, obtained at T_0_ and T_1_, were expressed as the decrease after licorice intake, in the absence (basal), or presence of DDS-NHOH. The activity of CA was assayed in the RBC cytosol as described in the Methods. The decrease following the licorice intake (ΔCA activity %) was calculated as: (1-activity T_1_/ activity T_0_)%. The monomerization of CA was assayed in diluted cytosol from 1 µL of packed RBCs, obtained as described in the Methods. The cytosol underwent Western blotting in non-reducing conditions and was immunostained with an anti-CA antibody. Densitometrical analysis of the CA bands was carried out and the sum of the 30 and 60 kDa bands was arbitrarily calculated as 100%, taking into account that amount of proteolytic 30 kDa bands accounts for half the larger [36]. Panel **b**: Total glutathione was determined according to the Tietze method [4], as described in the Methods. The total decrease of glutathione content after diamide or DDS-NHOH treatment (ΔGSH) was expressed as 1-GSH(Diam or DDS-NHOH)/GSH(Basal) [4,36].

**a**		**ΔCA activity %**	**CA 30 kDa (T_1_-T_0_ %)**
**CG**	**Diamide**	8.5 ± 7.0	−0.42 ± 0.79
	**DDS-NHOH**	50.7 ± 4.3 *	−12.08 ± 2.84 *
**PG**	**Diamide**	61.7 ± 16.0 †	−16.28 ± 4.27 †
	**DDS-NHOH**	79.1 ± 8.7 † *	−45.00 ± 6.94 † *
**b**		**ΔGSH (diamide)**	**ΔGSH (DDS-NHOH)**
**CG**	**T_0_**	0.08 ± 0.03	0.20 ± 0.01 *
	**T_1_**	0.08 ± 0.03	0.08 ± 0.03 ‡
**PG**	**T_0_**	0.54 ± 0.10 †	0.85 ± 0.08 † *
	**T_1_**	0.36 ± 0.08 † ‡	0.38 ± 0.08 † * ‡

Values are expressed as the mean ± SD of *n* = 12 healthy subjects (CG) and *n* = 18 patients (PG). Comparison from respective basal values: * *p* < 0.005, Student’s *t*-test for paired data. Comparison CG vs. PG: † *p* < 0.001, Student’s *t*-test for unpaired data. Comparison between T_0_ and T_1_ within each group: ‡ *p* < 0.001, Student’s *t*-test for paired data.

## Data Availability

The data presented in this study are available in the article.

## Supplementary Materials

The following are available online at https://www.mdpi.com/article/10.3390/ijms22168476/s1.
