# Supplementary material for: Endometriosis Susceptibility to Dapsone-Hydroxylamine-Induced Alterations Can Be Prevented by Licorice Intake: In Vivo and In Vitro Study
Source: Int J Mol Sci. 2021 Aug 6;22(16):8476. doi: 10.3390/ijms22168476 (PMC8395227; doi:10.3390/ijms22168476)
Supplement: Supplementary file 1 [file ijms-22-08476-s001.zip › ijms-1301256-supplementary.pdf]

Table S1. Clinical characteristics of CG and PG. Data are mean ± S.D.

Parameter	CG (*n* = 12)	PG (*n* = 18)
Age (yr)	34.9 ± 9.2	34.1 ± 8.5
Weight (kg)	58.9 ± 8.8	61.2 ± 6.8
BMI (kg/m^2)	22.0 ± 3.2	22.8 ± 1.7
Blood pressure (mmHg)		
Systolic	110.6 ± 9.2	116.2 ± 4.5
Diastolic	74.0 ± 10.3	78.2 ± 8.7
Menarche (median age, yr)	13.1	12.8
Cycle interval (last three months)	29.2 ± 2.0	27.8 ± 3.7
Previous pregnancies (n)	0	0
Smokers (n)	0	0
Hormone therapy	No	No
Any drug/chemicals	None	None
